# 肺癌合并间质性肺病的外科治疗

**DOI:** 10.3779/j.issn.1009-3419.2020.104.19

**Published:** 2020-05-20

**Authors:** 川 黄, 超 马, 青峻 吴, 鹏 焦, 耀光 孙, 文鑫 田, 瀚博 于, 文 黄, 永忠 王, 宏峰 佟

**Affiliations:** 100730 北京, 国家老年医学中心, 中国医学科学院老年医学研究院, 北京医院胸外科 Department of Thoracic Surgery, Beijing Hospital, National Center of Gerontology, Institute of Geriatric Medicine, Chinese Academy of Medical Sciences, Beijing 100730, China

**Keywords:** 肺疾病, 肺肿瘤, 肺切除术, 治疗结果, Lung diseases, Lung neoplasms, Pneumonectomy, Treatment outcome

## Abstract

**背景与目的:**

间质性肺病(interstitial lung disease, ILD)是一组主要累及肺间质和肺泡腔导致肺泡-毛细血管功能单位丧失的弥漫性肺疾病, 常导致限制性通气功能障碍和弥散功能障碍。ILD基础上肺癌发病率增高, 肺癌合并间质性肺病(lung cancer combined with ILD, LC-ILD)的手术风险明显增加。本研究旨在探讨LC-ILD外科治疗的安全性, 总结围术期诊治经验。

**方法:**

回顾性分析2012年1月-2019年12月北京医院胸外科收治的LC-ILD行肺切除术的患者资料, 总结其临床表现、影像、病理、手术安全性、围术期并发症和诊治经验。

**结果:**

本研究共纳入23例患者, 男性20例(87.0%), 平均年龄(69.1±7.8)岁, 吸烟者19例(82.6%)。ILD类型包括特发性肺纤维化14例(60.9%)、特发性非特异性间质性肺炎7例(30.4%)、结缔组织病相关ILD 2例(8.7%)。肺癌病理包括腺癌7例(30.4%)、小细胞癌7例(30.4%)、鳞癌6例(26.1%)、小细胞癌混合鳞癌1例(4.3%)、大细胞癌2例(8.7%)。手术入路包括经电视胸腔镜16例(69.6%)和前外侧开胸7例(30.4%), 切除方式包括肺叶切除13例(56.5%)、双肺叶切除1例(4.3%)和亚肺叶切除9例(39.1%)。术后90 d并发症11例(47.8%), 其中肺部并发症8例(34.8%), ILD急性加重(acute exacerbation of ILD, AE-ILD)4例(17.4%), 心房纤颤6例(26.1%), 急性左心功能不全1例(4.3%)。术后90 d死亡2例(8.7%), 死因均为AE-ILD。

**结论:**

LC-ILD以合并症多、肺功能差的高龄患者居多, 手术风险明显增高。术前应充分控制ILD病情, 术中尽量降低手术创伤, 术后应特别关注肺部并发症和AE-ILD。AE-ILD预后差, 治疗难度大, 糖皮质激素有助于改善病情, 早诊早治是治疗关键。

## Abstract (en)

**Background and objective:**

Interstitial lung disease (ILD) is a group of diffuse lung diseases that mainly involve the interstitial and alveolar cavities and result in loss of alveolar-capillary functional units, leading to restrictive ventilatory dysfunction and diffusion impairment. There was an increased incidence of lung cancer on the basis of ILD, and perioperative risk of patients with lung cancer combined with ILD (LC-ILD) was significantly increased. The aim of this study is to summarize the safety and experience of surgical treatment of LC-ILD.

**Methods:**

A retrospective analysis was performed on 23 patients with LC-ILD who underwent pneumonectomy in Beijing Hospital from January 2012 to December 2019, and their clinical manifestations, image feature, pathology, surgical safety, perioperative complications and treatment experience were summarized.

**Results:**

A total of 23 patients were included in this study, including 20 males (87.0%) with an average age of (69.1±7.8) years, and 19 cases (82.6%) were smokers. Of the ILD types, 14 cases (60.9%) were idiopathic pulmonary fibrosis, 7 cases (30.4%) were idiopathic nonspecific interstitial pneumonia, and 2 (8.7%) were interstitial lung disease associated with connective tissue diseases. The pathology of lung cancer included adenocarcinoma (30.4%, 7/23), small cell carcinoma (30.4%, 7/23), squamous cell carcinoma (26.1%, 6/23), small cell carcinoma mixed with squamous cell carcinoma (4.3%, 1/23) and large cell neuroendocrine carcinoma (8.7%, 2/23). Surgical approaches included video assisted thoracoscopy (69.6%, 16/23) and anterolateral thoracotomy (30.4%, 7/23), with lobectomy (52.2%, 12/23), double lobectomy (4.3%, 1/23), and sublobectomy (39.1%, 9/23). There were 11 cases (47.8%) of postoperative complications, including 8 cases (34.8%) of pulmonary complications, 4 cases (17.4%) of acute exacerbation of ILD (AE-ILD), 6 cases (26.1%) of atrial fibrillation, and 1 case (4.3%) of acute left ventricular dysfunction. The 90-day mortality is 8.7% (2/23) and the cause of death was acute exacerbation of ILD.

**Conclusion:**

Most of LC-ILD were elderly patients with multiple comorbidities and decreased pulmonary function, leading to significantly increased surgical risk. The ILD should be fully evaluated and controlled before surgery, intraoperative trauma should be minimized, and special attention should be paid to pulmonary complications and AE-ILD after surgery. Postoperative AE-ILD has a poor prognosis and glucocorticoids may be effective. Early diagnosis and treatment is the key to treatment of AE-ILD.

间质性肺病(interstitial lung disease, ILD)是一组主要累及肺间质和肺泡腔导致肺泡-毛细血管功能单位丧失的弥漫性肺疾病的总称, 表现为进行性加重的呼吸困难、限制性通气功能障碍和弥散功能障碍。ILD病因众多, 原发性ILD分类中以隶属于特发性间质性肺炎(idiopathic interstitial pneumonias, IIPs)的特发性肺纤维化(idiopathic pulmonary fibrosis, IPF)和特发性非特异性间质性肺炎(idiopathic nonspecific interstitial pneumonia, iNSIP)较常见, 而继发性ILD则以结缔组织病相关间质性肺病(ILD with connective tissue diseases, CTD-ILD)较常见。肺部慢性炎症和纤维化的基础上发生肺癌(lung cancer, LC)的风险明显增高, 肺癌合并间质性肺病(lung cancer combined with ILD, LC-ILD)在临床上越来越多见, 其治疗安全性和疗效也逐渐得到重视^[1, 2]^。ILD病程多为缓慢进展, 累及双肺, 肺功能明显减损, 肺切除术后肺部并发症的风险明显增高, 尤其是ILD急性加重(acute exacerbation of ILD, AE-ILD), 其早期诊断较困难, 缺乏有效治疗措施, 是导致术后死亡的重要原因^[3]^。目前国内有关LC-ILD手术治疗的报道较少, 为提高对该病的认识和治疗水平, 本研究总结2012年1月-2019年12月北京医院胸外科收治的LC-ILD行肺切除术的患者资料, 将其手术安全性和围术期管理经验报道如下。

## 1 材料与方法

### 1.1 入组对象

检索2012年1月-2019年12月北京医院胸外科收治的LC-ILD行肺切除术的患者, 其中诊断明确且资料完整者23例。

### 1.2 诊断标准

IIPs诊断标准参照2013年美国胸科学会和欧洲呼吸学会发表的IIPs分类意见和诊断标准^[4]^, IPF诊断标准参照IPF诊断和治疗中国专家共识^[5]^, CTD-ILD诊断标准参照2018中国CTD-ILD诊断和治疗专家共识^[6]^, LC均经手术病理证实, 按照第8版国际肺癌肿瘤原发灶-淋巴结-转移(tumor-node-metastasis, TNM)分期。

### 1.3 手术安全性和围术期并发症

查阅病历并进行门诊或电话随访, 统计手术时间、术中出血量、术中输血、中转开胸、胸腔引流量、胸管留置时间、术后住院时间等手术安全性指标。统计术后90 d内并发症和死亡, 并发症的定义参照美国胸外科医师协会普胸外科数据库^[7]^, AE-ILD的定义参照2016年国际多学科定义和诊断标准^[8]^。

### 1.4 统计方法

采用SPSS 22.0软件进行统计分析, 正态分布的计量资料以均数±标准差表示, 非正态分布的计量资料以中位数表示。

## 2 结果

### 2.1 一般资料

男性20例(87.0%), 女性3例(13.0%), 平均年龄(69.1±7.8)岁。临床症状包括咳嗽21例(91.3%)、咳痰11例(47.8%)、胸闷气短9例(39.1%)、活动耐力下降8例(34.8%)。吸烟者19例(82.6%), 吸烟指数20包年-150包年。有术前合并症者20例(87.0%)。中位ILD病史时间为36个月(0.5个月-240个月), ILD类型包括IIPs 21例(91.3%), 其中IPF 14例, iNSIP 7例, 2例IPF曾有10余年有毒化学物质和粉尘接触史。CTD-ILD 2例(8.7%), 其中干燥综合征继发ILD 1例, 系统性硬化症继发ILD 1例。LC病理包括腺癌7例(30.4%)、鳞癌6例(26.1%)、小细胞癌7例(30.4%)、小细胞癌混合鳞癌1例(4.3%)、大细胞神经内分泌癌2例(8.7%)。患者一般资料、术前合并症、ILD类型见表 1, 肿瘤部位、病理类型和分期见表 2。

**1:** 23例患者的一般资料 Clinical characteristics of 23 patients

Category	Data
Gender	
Male	20(87.0%)
Female	3(13.0%)
Age(yr), Mean±SD(range)	69.1±7.8(53-80)
Body mass index(kg/m^2^), Mean±SD(range)	25.4±3.1(19.6-30.5)
Smoking history	
Yes	19(82.6%)
No	3(17.4%)
Smoking index by pack-years, Md(range)	50(20-150)
Comorbidity	20(87.0%)
Hypertension	9(39.1%)
Diabetes mellitus	7(30.4%)
Coronary artery disease	7(30.4%)
Chronic obstructive pulmonary disease	4(17.4%)
Autoimmune disease	2(8.7%)
History of other tumors	2(8.7%)
Type of ILD	
IPF	14(60.9%)
iNSIP	7(30.4%)
CTD-ILD	2(8.7%)
History of ILD(mo), Md(range)	36(0.5-240)
Md: median; SD: standard deviation; ILD: interstitial lung disease; IPF: idiopathic pulmonary fibrosis; iNSIP: idiopathic nonspecific interstitial pneumonia; CTD-ILD: connective tissue diseases related interstitial lung disease.	

**2:** 23例患者的肿瘤特点 Tumor characteristics of 23 patients

Category	Data
Site of tumor	
Right upper lobe	7(30.4%)
Right middle lobe	1(4.3%)
Right lower lobe	5(21.7%)
Left upper lobe	6(26.1%)
Left lower lobe	4(17.4%)
Location	
Central type	5(21.7%)
Peripheral type	18(78.3%)
Pathological type	
Adenocarcinoma	7(30.4%)
Squamous carcinoma	6(26.1%)
Small cell carcinoma	7(30.4%)
Small cell carcinoma combined with squamous carcinoma	1(4.3%)
Large cell neuroendocrine carcinoma	2(8.7%)
TNM stage	
Ⅰ	12(52.2%)
Ⅱ	3(13.0%)
Ⅲ	5(21.7%)
Ⅳ	3(13.0%)
TNM: tumor-node-metastasis.	

### 2.2 肿瘤标记物

血液肿瘤标记物升高者15例(65.2%), 其中癌胚抗原升高8例, 细胞角蛋白19片段升高10例, 神经元特异性烯醇化酶升高3例, 鳞状上皮细胞癌抗原升高2例, CA125升高3例, CA199升高2例, CA153升高3例, CA724升高1例。

### 2.3 肺功能和动脉血气

通气功能障碍13例(56.5%), 其中阻塞性通气功能障碍5例, 限制性通气功能障碍5例, 混合性通气功能障碍3例。弥散功能障碍16例(69.6%)。动脉血氧分压(PaO_2_) < 80 mmHg者9例(39.1%), 二氧化碳分压(PaCO_2_)均在正常范围。肺功能和动脉血气指标见表 3。

**3:** 23例患者的临床症状和肺功能 Symptoms and pulmonary function of 23 patients

Category	Data
Symptoms	
Cough	21 (91.3%)
Expectoration	11 (47.8%)
Chest distress	9 (39.1%)
Shortness of breath after activities	8 (34.8%)
Pulmonary function	
Obstructive ventilation dysfunction	5 (21.7%)
Restrictive ventilation dysfunction	5 (21.7%)
Mixed ventilation dysfunction	3 (13.0%)
Diffusion dysfunction	16 (69.6%)
Pulmonary function parameters	Mean±SD (range)
FEV_1_ (%pred)	78.6±17.3 (50.0-123.0)
FVC (%pred)	83.4±15.4 (53.0-112.0)
FEV_1_/FVC (%pred)	72.6±11.8 (44.6-91.4)
MVV (%pred)	77.4±20.1 (40.8-134.0)
VC (%pred)	82.5±14.2 (56.0-109.0)
TLC (%pred)	81.7±12.2 (62.6-107.0)
DLCO (%pred)	66.0±17.5 (21-92)
Arterial blood gas analysis	Mean (range)
PaO_2_ (mmHg)	80.0±8.0 (64-97)
PaCO_2_ (mmHg)	38.3±3.7 (33-45)
SaO_2_ (%)	95.9±1.9 (90-98)
SD: standard deviation; FEV_1_: forced expiratory volume in 1 second; FVC: forced vital capacity; MVV: maximal voluntary ventilation; VC: vital capacity; TLC: total lung capacity; DLCO: carbon monoxide diffusing capacity; PaO_2_: arterial oxygen pressure; PaCO_2_: arterial carbon dioxide pressure; SaO_2_: arterial oxygen saturation.	

### 2.4 影像特征

ILD的胸部CT表现为双肺多发网格影、斑片磨玻璃影、蜂窝影或囊状影, 常伴小叶间隔增厚, 多沿双肺胸膜下分布, 亦可累及大部分肺野。本组ILD最常累及双肺下叶外周, 沿胸膜下分布为主者19例(82.6%), 双肺弥漫分布者4例(17.4%)。LC胸部CT表现为周围型占位18例(78.3%), 中央型占位5例(21.7%)。典型CT图像见图 1。

**1:**
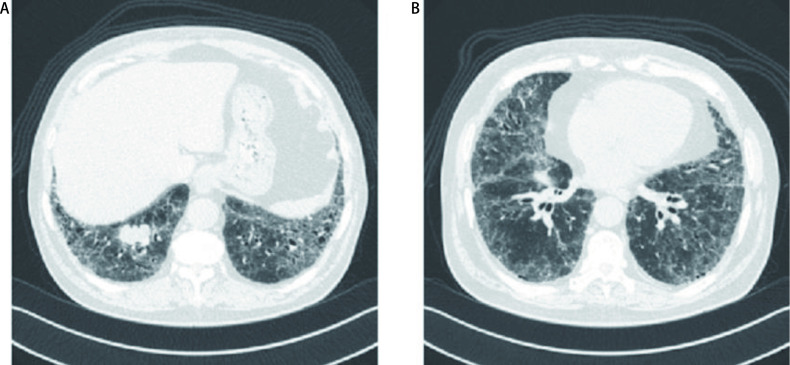
74岁, 男性, IPF合并右肺下叶小细胞癌。胸部CT显示双肺多发网格影、蜂窝影及磨玻璃影, 伴小叶间隔增厚, 以胸膜下和下叶为著。 A 74-years-old male, IPF combined with small cell lung cancer in the right lower lobe. Chest CT showed multiple reticular shadows, honeycomb shadows, and ground glass opacities in bilateral lungs, accompanied by thickening of interlobular septum. IPF lesions mainly distributed along subpleural area and inferior lobe. CT: computed tomography; IPF: idiopathic pulmonary fibrosis.

### 2.5 治疗方式

ILD治疗以氧疗为主, 术前长期使用糖皮质激素3例, ILD控制稳定者21例(91.3%), 逐渐进展者2例(8.7%)。手术入路包括经电视胸腔镜16例(69.6%)和前外侧开胸7例(30.4%), 切除方式包括肺叶切除13例(包括支气管袖状切除1例, 左肺下叶切除术后间隔3月再行右肺楔形切除1例), 右肺中下叶切除1例, 亚肺叶切除9例(包括楔形切除8例, 肺段切除1例)。手术时间、出血量、胸腔引流量、胸管留置时间和术后住院时间见表 4。

**4:** 23例患者的围术期结果和并发症 Perioperative outcomes and complications of 23 patients

Perioperative outcomes	Data
Surgical procedures	
Lobectomy	13 (56.5%)
Bilobectomy	1 (4.3%)
Sublobectomy	9 (39.1%)
Operation time (min), Mean±SD (range)	140.0±90.3 (50-440)
Intraoperative blood loss (mL), Md (range)	100 (20-700)
Intraoperative blood transfusion	3 (13.0%)
Conversion to thoracotomy	1 (4.3%)
Thoracic drainage duration (d), Md (range)	3 (2-13)
Thoracic drainage volume (mL), Mean±SD (range)	1257.8±914.7 (30-3600)
Postoperative hospital stay (d), Md (range)	14 (5-90)
Postoperative complications	11 (47.8%)
Pulmonary complications	8 (34.8%)
AE-ILD	4 (17.4%)
Atrial arrhythmia	6 (26.1%)
Acute left ventricular dysfunction	1 (4.3%)
90-day mortality	2 (8.7%)
Md: median; SD: standard deviation; AE-ILD: acute exacerbation of interstitial lung disease.	

### 2.6 围术期并发症

术后90 d并发症11例(47.8%), 死亡2例(8.7%), 死亡原因均为AE-ILD, 见表 4。肺部并发症8例(34.8%), 其中肺部感染4例, 肺持续漏气超过5天者3例, 胸腔积液需穿刺引流者1例, 呼吸衰竭6例。心房纤颤需药物治疗者6例(26.1%), 急性左心功能不全1例(4.3%)。AE-ILD 4例(17.4%), 发生于术后第2-5 d, 首发症状均为不明原因的咳嗽、憋喘, 常规氧疗下血氧饱和度难以维持, 快速进展至严重呼吸困难和呼吸衰竭, 胸部影像显示双肺新增大片磨玻璃影或实变影, 呼吸道病原学筛查无明确感染证据, 气管插管行机械通气2例, 无创呼吸机辅助通气1例, 4例确诊后均使用糖皮质激素治疗, 好转2例, 死亡2例。1例术后出现AE-ILD并治疗成功患者的胸部影像变化见图 2。

**2:**
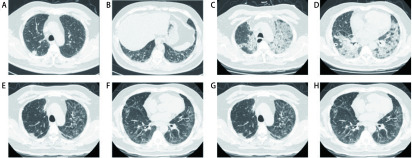
60岁, 男性, IPF合并右肺上叶小细胞癌。A、B：术前胸部CT显示双肺多发网格影、磨玻璃影及索条影, 伴小叶间隔增厚, 以双下肺为著; C、D：术后第3天出现IPF急性加重, 双肺新增多发小叶间隔增厚、磨玻璃影、网格影及索条影, 较术前明显进展; E、F：糖皮质激素治疗14 d后, 双肺弥漫斑片状磨玻璃影较前明显吸收减少; G、H：糖皮质激素治疗42 d后, 双肺间质性炎症吸收消散, 患者顺利出院。 A 60-years-old male, IPF combined with small cell lung cancer in the right upper lobe. A, B: Preoperative chest CT showed multiple reticular shadows, ground glass opacities and strip shadows, accompanied by thickening of interlobular septum, which mainly located in bilateral inferior lobe. C, D: Acute exacerbation of IPF occurred on the 3^rd^ day after surgery. Chest CT showed the newly appeared multiple reticular shadows, ground glass opacities and strip shadows, accompanied by thickening of interlobular septum. The degree of IPF was significantly worse than preoperative lesions. E, F: After 14 days of glucocorticoid treatment, the diffused ground glass opacities in bilateral lungs were significantly absorbed and decreased. G, H: After 42 days of glucocorticoid treatment, bilateral interstitial pneumonia was absorbed and the patient was discharged smoothly.

## 3 讨论

ILD多呈缓慢进展, 表现为咳嗽、活动耐力下降和进行性加重的呼吸困难, 胸部高分辨率CT(high resolution computed tomography, HRCT)是筛查ILD的首选方法, 常表现为肺部磨玻璃影、网格影或蜂窝影, 可伴小叶间隔增厚或牵拉性支气管扩张, 多沿肺下野外周带分布, 严重者呈双肺弥漫分布。本组ILD沿胸膜下分布者19例(82.6%), 术后肺部并发症率为21.1%(4/19), 其中AE-ILD 3例、肺部感染1例, 3例好转, 1例死亡。而ILD呈双肺弥漫分布者4例(17.4%), 肺部并发症率达50%(2/4), 其中1例术后第2天出现AE-ILD, 经糖皮质激素、机械通气治疗后无法改善, 死于呼吸衰竭; 另1例术后第52天因感冒受凉后出现严重肺部感染, 经长期抗感染、无创呼吸机辅助通气, 最终病情好转。因此, 术前行HRCT评估ILD肺部受累程度和范围对预测术后并发症有重要意义, 术后应重点关注肺部并发症(尤其是AE-ILD), 即使术后短期内恢复顺利, 出院后也应继续随访至少3个月, 谨防肺部病情反复。

ILD患者罹患LC的风险明显增高, 文献^[9]^报道LC-ILD的发病率达10%-20%, 风险比(odds ratio, OR)达3.52(95%CI: 1.94-6.37), 其中CTD-ILD患者中LC发病率为5%左右, IPF患者中LC的发病率达13%-20%, OR达4.99(95%CI: 3.03-8.22)。因此, ILD随诊过程中应关注有无咯血、肿瘤标记物升高, 胸部影像发现肺部占位即应高度怀疑LC-ILD。LC常发生于ILD病变较重的肺野, 肺间质反复慢性炎症、纤维化基础上肺泡上皮细胞增生修复、纤维变性是诱发癌变的重要因素, 高龄、男性、吸烟、职业和环境暴露、胃食管反流病、病毒感染等因素可增加癌变风险。本组LC病理类型以小细胞癌(34.8%)最多, 其次是腺癌(30.4%)和鳞癌(26.1%), 5例(21.7%)为中央型LC, 14例(60.9%)需行肺叶甚至双肺叶切除。70岁以上高龄患者近60%, 男性(87.0%)、吸烟者(82.6%)比例高, 近90%患者术前合并伴随疾病, 其中有慢性支气管炎和肺气肿、冠心病等严重合并症者近50%。82.6%(19/23)患者术前有肺通气功能障碍或弥散功能障碍, 肺功能下降者术后肺部并发症率达42.1%(8/19), 肺功能正常者术后均未出现肺部并发症。因此, 充分评估伴随疾病和肺功能, 筛查高危因素, 做好应对措施, 是LC-ILD术前管理的重点。

ILD病因众多, 已知病因的ILD约占35%, 诱因包括职业或环境因素、药物因素、病毒或细菌感染、结缔组织病等, 其中类风湿性关节炎、系统性硬化症、系统性红斑狼疮、干燥综合征、多发性肌炎/皮肌炎等结缔组织病(connective tissue diseases, CTD)均可诱发ILD, 即CTD-ILD。本组共2例CTD-ILD, 其中1例为53岁女性, 系统性硬化症病史15年, ILD病史8年, 曾行糖皮质激素治疗1年将病情控制稳定后停药, 术前长期使用N-乙酰半胱氨酸控制病情, 术后病理为腺癌。另1例为74岁男性, 干燥综合征和ILD病史4年, 随诊期间发现右肺下叶结节且逐渐增大, 术前未使用激素或免疫抑制剂, 术后病理为小细胞癌, 术后第3天出现AE-ILD, 虽经糖皮质激素、抗感染和机械通气治疗, 但双肺间质性炎症始终无法改善, 死于呼吸衰竭。

另有约65%的ILD病因不明, 故命名为特发性, 主要类别为IIPs, 包括IPF、iNSIP、呼吸性细支气管炎伴ILD、脱屑性间质性肺炎、隐原性机化性肺炎、急性间质性肺炎等若干类型^[4]^, 其中以IPF和iNSIP较常见, 二者的典型影像和病理表现分别为普通型间质性肺炎(usual interstitial pneumonia, UIP)和非特异性间质性肺炎(nonspecific interstitial pneumonia, NSIP)。本组IIPs共21例(91.3%), 其中2例患者有长期接触有毒化学物质和染料的工作史, 例行体检时发现ILD。14例IPF胸部CT表现为以胸膜下、基底部分布为主的网格影和蜂窝影, 3例伴小叶间隔增厚, 沿胸膜下分布者11例, 双肺弥漫分布者3例, 术后病理为鳞癌5例、腺癌3例、小细胞癌4例、大细胞神经内分泌癌2例。另7例iNSIP胸部CT则表现为双肺胸膜下磨玻璃斑片影, 部分伴胸膜下网格状改变, 术后病理为小细胞癌3例、腺癌3例、鳞癌1例。本组IIPs术后并发症率高达47.6%(10/21), 肺部并发症7例, 心血管并发症7例, AE-ILD 3例(14.3%, 3/21), 其中2例好转, 1例死亡。

因此, LC-ILD患者诊治过程中应注重询问自身免疫性疾病、特殊职业和环境接触史等相关病史, 结合病史和影像特征判断ILD类型。术前充分评估伴随疾病和全身脏器功能, 掌握患者的糖皮质激素、免疫抑制剂等用药史, 围术期可联合内科指导用药, 将自身免疫性疾病和ILD控制稳定, 尽量避免因药物减量和停药诱发的病情加重, 从而提高围术期安全性。

AE-ILD是LC-ILD围手术期最严重的并发症之一, 支气管镜检查和镜下治疗、肺切除、胸部放疗、部分药物等均可诱发AE-ILD, 肺切除术后IPF急性加重(acute exacerbation of IPF, AE-IPF)发生率达12%-27%, 典型症状为快速进展的呼吸困难, 肺部可闻及吸气末爆裂音(velcro啰音), HRCT表现为在UIP基础上出现新的磨玻璃影和(或)实变影, 常需大剂量糖皮质激素治疗或联合免疫抑制剂治疗, 死亡率依然高达30%-100%^[10]^。本组AE-ILD发生率为17.4%(4/23), 其中3例ILD病变沿双肺胸膜下分布、1例累及双侧大部分肺野, 病理均为小细胞癌, 3例行肺叶切除, 1例行楔形切除。1例为CTD-ILD在术后第3天出现急性加重, 另3例为IPF在术后第2-5天出现急性加重, 早期均表现为憋喘、呼吸困难和血氧饱和度下降, 快速进展至呼吸衰竭, 死亡率达50%(2/4)。值得注意的是, 2例治疗失败者属早年诊治病例, 当时我们对AE-ILD的诊治经验尚不足, 术后早期出现憋喘、低氧血症时未立即行HRCT确立诊断, 贻误最佳治疗时机, 分别于加重后第2天、第19天才加用糖皮质激素, 其中1例经激素治疗5 d后病情明显改善, 遂将激素快速减量, 随后双肺间质性炎症再次加重, 恢复激素用量后仍无效, 最终死于呼吸衰竭。前车之鉴, 此后2例LC-ILD患者术后出现难以解释的呼吸困难时, 加重当日即行HRCT发现肺间质炎症明显进展, 在完善病原学筛查和抗生素治疗的同时, 早期加用足量糖皮质激素, 并维持至病情彻底稳定后才缓慢减量, 最终治疗均成功。

男性、既往曾发生AE-IPF、肺切除范围大、胸部CT表现为UIP、血清涎液化糖链抗原-6 > 1, 000 U/mL、FEV_1_%≤80%、肺弥散功能降低等均是肺切除术后AE-IPF的高危因素, 肺叶、双肺叶、全肺切除术后AE-IPF发生率明显高于楔形切除^[11, 12]^。本组肺叶切除术后AE-ILD发生率达21.4%(3/14), 明显高于亚肺叶切除者(14.3%, 1/7)。对于ILD病史久、肺受累范围广、病情控制不稳定的高危病例, 需谨慎选择手术时机和方式, 尤其是肺功能明显下降、伴随疾病众多的高龄患者, 首选微创术式。IPF预后很差, 确诊后中位生存期仅2年-3年, 因此, LC-ILD患者制定手术方案时, 应兼顾肿瘤学根治原则与全身病情, 谨慎选择肺切除范围, 对于ILD病情轻、心肺功能良好者, 可首选根治性肺叶切除, 但对于ILD病情严重、心肺功能明显下降者, 则宜选择肺段或楔形切除, 避免因创伤过大导致围术期风险显著升高。

部分肺癌治疗药物也可诱发AE-ILD, 细胞毒性药物诱发AE-ILD发生率达13%-22%^[13, 14]^, 酪氨酸激酶抑制剂诱发AE-ILD发生率达5%-10%^[15, 16]^, 免疫检查点抑制剂相关ILD发生率达3.5%-16.9%, 其中4级和致死性ILD的比例达19%^[17, 18]^, 因此LC-ILD药物治疗期间也应密切关注ILD病情变化, 警惕AE-ILD发生。新型抗纤维化药物吡非尼酮和尼达尼布可延缓肺纤维化进程, 降低肺纤维化急性进展风险^[19]^, 围术期使用吡非尼酮可降低肺切除术后AE-IPF发生率^[20]^。

综上所述, 在LC-ILD患者的诊治过程中, 术前应全面评估伴随疾病和高危因素, 合并自身免疫性疾病者应控制稳定, 合并冠心病、心功能不全等心血管疾病者应改善心功能和预防心血管事件, 合并肺部病毒或细菌感染者应针对性用药, 合并胃食管反流者应使用质子泵抑制剂以降低误吸风险, 长期吸烟者应戒烟并加强气道管理, 改善全身脏器功能, 并将ILD病情控制稳定, 从而降低治疗风险。LC-ILD应严格把握手术适应证, 不宜为追求根治而盲目扩大切除范围, 而应在肿瘤根治原则和降低手术创伤之间谨慎权衡, 提高手术安全性。围术期和术后随访期间应严密监测呼吸道症状和胸部影像改变, 重点关注肺部并发症和AE-ILD, 疑诊者尽早行HRCT和病原学筛查, 早期诊断是AE-ILD治疗的关键, 早期、足量、足疗程糖皮质激素有助于改善病情, 治疗期间应谨防病情反复。通过术前严格把控, 术中精细管理, 术后早诊早治, 最终提高LC-ILD患者的手术安全性和疗效。
